# Hierarchically-structured silver nanoflowers for highly conductive metallic inks with dramatically reduced filler concentration

**DOI:** 10.1038/srep34894

**Published:** 2016-10-07

**Authors:** Muhammed Ajmal C., Faseela K. P., Swati Singh, Seunghyun Baik

**Affiliations:** 1 Department of Energy Science, Sungkyunkwan University, Suwon, 16419, Republic of Korea; 2 School of Mechanical Engineering, Sungkyunkwan University, Suwon, 16419, Republic of Korea; 3 Center for Integrated Nanostructure Physics, Institute for Basic Science (IBS), Suwon, 16419, Republic of Korea

## Abstract

Silver has long been employed as an electrically conductive component, and morphology-dependent properties have been actively investigated. Here we present a novel scalable synthesis method of flower-shaped silver nanoparticles (silver nanoflowers, Ag NFs). The preferential affinity of citrate molecules on (111) surface of silver enabled spontaneous anisotropic growth of Ag NFs (bud size: 250~580 nm, single crystalline petal thickness: 9~22 nm) with high reproducibility and a high yield of >99.5%. The unique hierarchical structure resulted in coalescence of petals over 80~120 °C which was practically employed in conductive inks to construct percolation pathways among Ag NFs. The ink with only 3 wt% of Ag NFs provided two orders of magnitude greater conductivity (1.008 × 10^5^ Scm^−1^), at a low curing temperature of 120 °C, compared with the silver nanoparticle ink with a much higher silver concentration (50 wt%). This extraordinary property may provide an excellent opportunity for Ag NFs for practical applications in printable and flexible electronics.

Silver has long been employed as an electrically conductive component for electronic devices. Various morphologies of silver particles have been intensively investigated since the electrical properties are governed by their size and structure^1^−^4^. Microscale silver flakes or spherical particles were mixed with polymer matrix to produce viscous conductive pastes^5^,^6^. Silver nanoparticles were employed for low-viscosity conductive inks due to the better dispersion and low sintering temperature^7^,^8^. Silver nanowires with high aspect ratios were developed for transparent conductive films since percolation could be achieved at a lower concentration^9^,^10^,^11^. The hybrid silver nanoparticle-nanowires^12^, fractal dendrites^2^, and self-assembled hierarchical structures^13^ were studied to achieve low percolation threshold and high electrical conductivity by multi-dimensional geometries.

The surface wettability and optical properties of silver were also investigated using flower-shaped morphologies^14^,^15^,^16^,^17^,^18^,^19^,^20^. Superhydrophobicity was induced by increasing surface roughness of silver particles (0.5~3 μm) through galvanic reaction^16^,^17^. The improved sensitivity of surface-enhanced Raman scattering was reported using particles (0.5~4 μm) synthesized by the solution process due to the strong coupling between surface plasmon resonance of metals and incident light^14^,^15^,^18^,^19^,^20^. More recently, flower-shaped silver particles (~800 nm) were employed to achieve high electrical conductivity of stretchable polymer matrix fibers^21^.

Here we present a scalable synthesis method of flower-shaped silver nanoparticles (silver nanoflowers, Ag NFs). The morphology could be controlled with high reproducibility by changing reagent concentrations (bud size: 250~580 nm, petal thickness: 9~22 nm). The preferential affinity of citrate molecules on (111) surface of silver enabled spontaneous anisotropic growth of Ag NFs. The synthesis could be easily upscaled with a high yield of >99.5%. The petals of Ag NFs actively coalesced at a low curing temperature of 80~120 °C dramatically enhancing electrical connectivity among Ag NFs. The aqueous dispersion of Ag NFs was excellent with a small addition of hydroxypropyl methyl cellulose (HPMC, 0.6 wt%). Two orders of magnitude greater electrical conductivity (1.008 × 10^5^ Scm^−1^) was achieved even at a much lower silver concentration (3 wt%), when the ink pattern was cured at 120 °C, compared with a commercial silver nanoparticle ink (2127 Scm^−1^, Ag = 50 wt%). This demonstrated an excellent electrical conductive pathway construction by low-temperature coalescence of Ag NFs.

## Synthesis of Ag NFs

**Figure 1:**
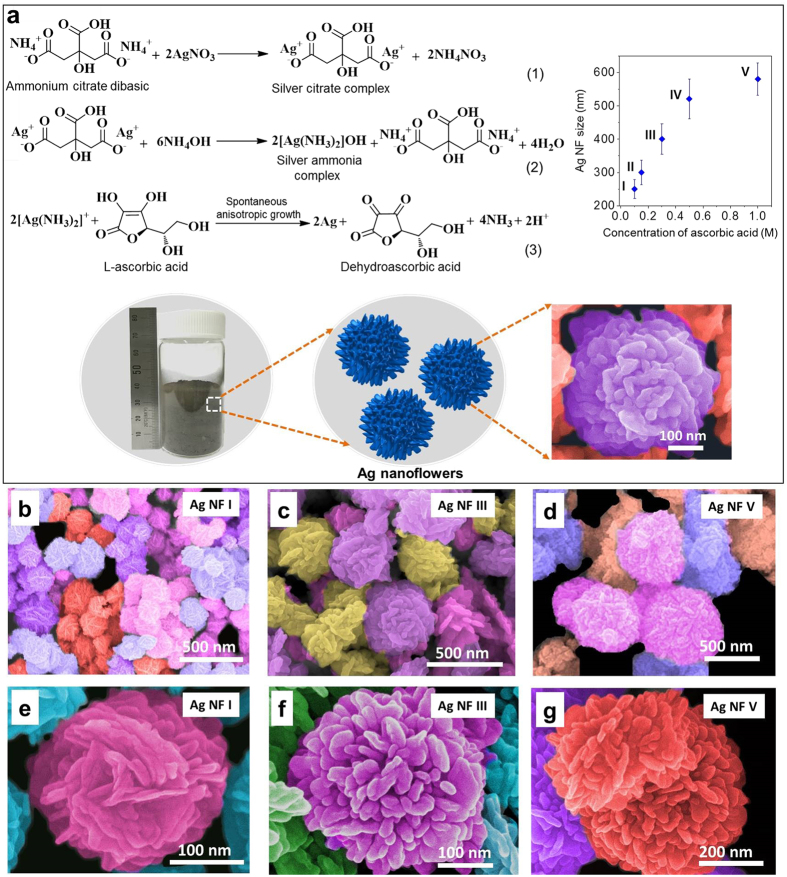
Synthesis of Ag NFs. (**a**) Schematic of the synthesis process. The bud size of Ag NFs is shown as a function of the ascorbic acid concentration (inset). (**b–g**) Pseudo-colored and magnified SEM images of Ag NF I, III, and V.

Figure 1a shows a schematic of the Ag NF synthesis process. The water-based, surfactant-free process enabled scalable synthesis of Ag NFs with high reproducibility. A detailed procedure is explained in Methods, and a brief description is provided below. Firstly, aqueous solutions of silver nitrate (0.3 M) and ammonium citrate dibasic (0.15 M) were mixed at 50 °C. The ammonium citrate acted as an initial complexation agent to control anisotropic growth of silver^22^. The citrate molecules preferentially bind to (111) plane of face-centered cubic (fcc) structure of silver^22^. This promoted anisotropic growth maximizing the surface area of (111) plane as will be discussed later. The citrate-guided growth has advantage over surfactant-aided anisotropic growth^14^,^20^,^23^,^24^ since the remnant surfactant on the surface hinders electrical connectivity among particles. In the next step, aqueous ammonia (14.8 M) was added to form [Ag (NH_3_)_2_]^+^ion complex, and boric acid (0.5 M) was added to maintain pH of the mixture^17^. Finally, the addition of ascorbic acid (0.1~1 M) as a reducing agent^15^,^18^,^25^ resulted in spontaneous anisotropic growth of Ag NFs and immediately turned color of the solution into dark brown. The hydroxyl proton situated at the end of the vinyl group is extraordinarily acidic compared with the common alcohol hydroxyl group because of extra stability of the conjugate base of ascorbic acid^25^. The transfer of two electrons from the ascorbic acid resulted in reduction of silver ions. Figure 1a shows 100 g of Ag NFs synthesized in multiple batches. The synthesis was scalable, and the amount of Ag NFs obtained in one batch was only limited by the amount of reagents. The maximum amount of Ag NFs synthesized in one batch was 50 g in this study. The yield, defined by the mass ratio of Ag NFs and Ag ions^26^, was greater than 99.5% when chemically equivalent or greater amount of reducing agent was provided.

Ag NFs had hierarchical structures with large buds (250~580 nm) and thin petals (9~22 nm) as will be discussed shortly with scanning electron microscopy (SEM) and high-resolution transmission electron microscopy (HRTEM) analysis. The average bud size increased significantly as the concentration of ascorbic acid (0.1~1 M) increased (Fig. 1a inset). The size distributions of Ag NFs and their petals are also provided in Supplementary Figs 1 and 2. The Ag NFs synthesized at five different ascorbic acid concentrations were named as Ag NF I, II, III, IV, and V. A greater amount of ascorbic acid resulted in a more spontaneous reduction and larger bud size. The specific surface area of Ag NFs, measured by Brunaur-Emmet-Teller (BET) adsorption isotherm^2^,^21^, also increased as the concentration of ascorbic acid increased (2.08, 3.82, and 5.15 m^2^g^−1^ for Ag NF I, III, and V). However, the flower structure with thin petals could not be maintained when the concentration of ascorbic acid was too high (2 M). The change in reaction time (10 sec ~120 min) did not affect the bud size of Ag NF III (0.3 M of AgNO_3_ and 0.3 M of ascorbic acid) confirming the spontaneous anisotropic growth (Supplementary Fig. 3a). The change in temperature below 80 °C did not affect the bud size of Ag NF III (Supplementary Fig. 3b,c). However, the flower petal structure changed when the reaction temperature was greater than 90 °C.

Figure 1b–g show low and high magnification pseudo-colored SEM images of Ag NF I, III, and V. Raw SEM images of Ag NF I, II, III, IV, and V are provided in Supplementary Figs 4 and 5. The increasing bud size with increasing ascorbic acid concentration is clearly shown. However, there was only a little increase (9~22 nm) in the thickness of petals. The thin petals resulted in active coalescence among Ag NFs upon curing as will be discussed later. The ultraviolet-visible (UV-Vis) absorption spectra revealed the out-of-plane surface quadrupole plasmon resonance at 340 nm for Ag NFs due to the thin petal structure (Supplementary Fig. 6)^1^,^2^. In contrast, spherical silver nanoparticles (~70 nm) exhibited a peak at 405 nm corresponding to the characteristic plasmon resonance of spherical silver nanoparticles^27^,^28^. The energy dispersive X-ray analysis revealed major peaks corresponding to metallic silver^29^ (Supplementary Fig. 7).

## Crystallinity of Ag NFs

**Figure 2:**
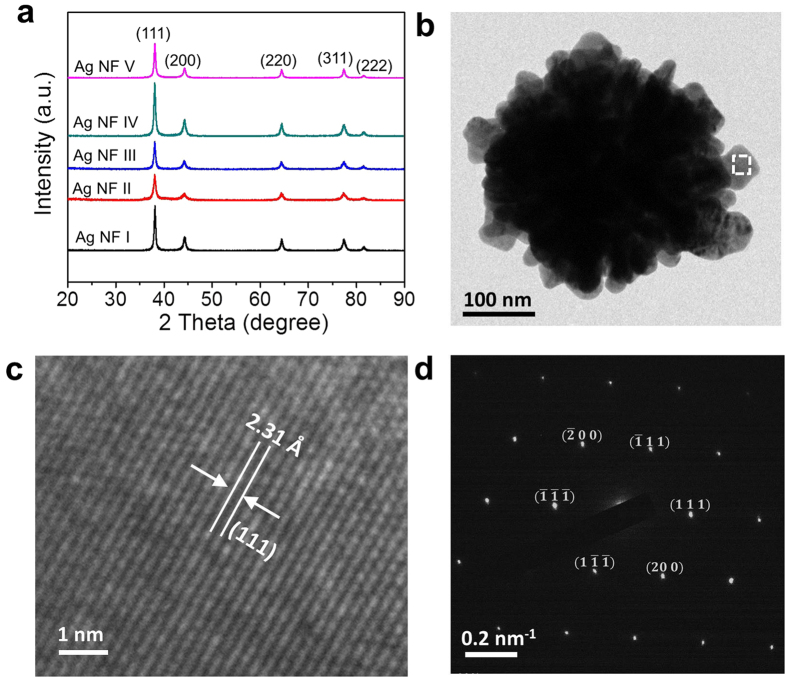
Facet analysis of Ag NFs. (**a**) XRD spectra of Ag NFs. (**b–d**) HRTEM analysis of Ag NF III. A lattice-resolved image and selected-area electron diffraction pattern of the square region are also provided. The lattice fringe distance of 2.31 Å is consistent with the d-spacing of (111) plane of single crystalline face-centered-cubic silver.

The crystallinity of Ag NFs was investigated by X-ray diffraction (XRD) and HRTEM analysis. Figure 2a shows XRD patterns of Ag NFs. The observed peaks corresponded to (111), (200), (220), (311), and (222) facets of fcc structure of silver (JCPDS 65-2871). The (111)/(200) intensity ratio (3.17~3.56) was greater than the standard file value (2.1) indicating abundant (111) facet^30^,^31^. The HRTEM analysis also confirmed the dominant (111) facet of petals (Fig. 2b–d). Figure 2c shows a lattice-resolved HRTEM image of a petal (marked square area in Fig. 2b). The lattice fringe distance of 2.31 Å was consistent with the d-spacing between (111) planes in single-crystalline fcc structure of silver (2.35 Å from JCPDS 65-2871). The selected area electron diffraction (SAED) analysis of the square region in Fig. 2b also revealed that each petal was a single-crystalline protrusion (Fig. 2d). The entire Ag NF was poly-crystalline with numerous petals with different orientations. The preferential affinity of citrate molecules on (111) plane increased (111) facet in petal surface planes^22^. Besides, the surface energy of (111) facet is lower than other planes maximizing the surface area of (111) facet during the thermodynamically-favorable synthesis^32^.

## Coalescence Physics of Ag NFs Upon Curing

**Figure 3:**
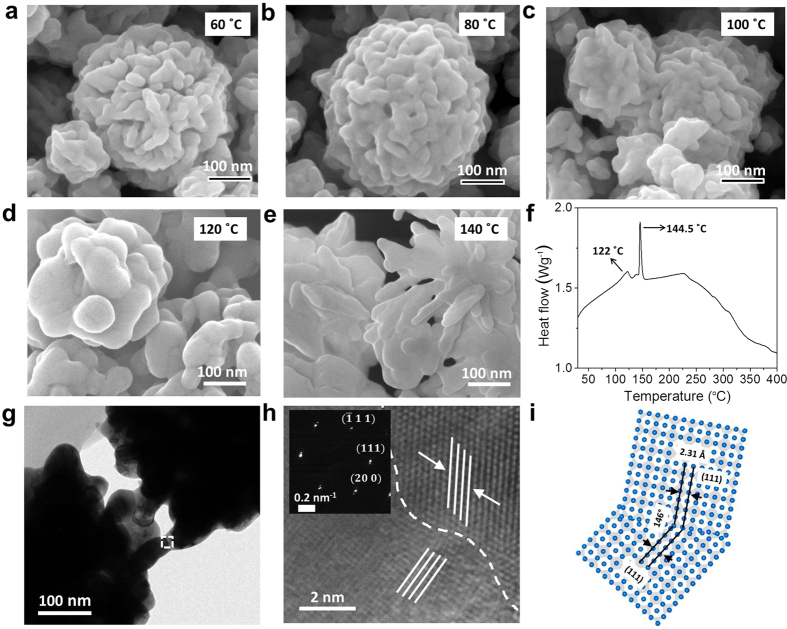
Coalescence physics of Ag NF III. (**a–e**) SEM images after curing at different temperatures (60, 80, 100, 120, and 140 °C) for 30 min. The images show different Ag NFs in the powder mixture. (**f**) DSC analysis. (**g–h**) HRTEM images of coalesced Ag NFs after curing at 120 °C for 30 min. A lattice-resolved image and SAED analysis of the interface between two-coalesced petals (square region) are provided. **i,** Schematic of the merged Ag NF petals.

The coalescence of Ag NFs takes place with adequate thermal energy. Figure 3a–e show morphology change of Ag NF III cured at different temperatures (60~140 °C) for 30 minutes in ambient air condition. There was no change when the curing temperature was 60 °C (Fig. 3a). However, the petals with nanoscale thickness (~12 nm) started to deform at a low curing temperature of 80 °C (Fig. 3b). The morphology change at 80 °C became more evident when the curing time was further increased up to 120 min whereas there was no noticeable change when the curing temperature was 60 °C (Supplementary Fig. 8). The sharp flower structure was strikingly deformed at 120 °C due to the active morphology change and coalescence of petals (Fig. 3d). The Ag NFs were completely coalesced and somewhat flattened when the curing temperature was further increased to 140 °C (Fig. 3e)^21^. In contrast, there was no active coalescence of spherical silver nanoparticles (~70 nm) even at a higher curing temperature of 150 °C (Supplementary Fig. 9)^21^, demonstrating excellent coalescence behavior of 10~15 nm thick petals. Unlike evenly surrounded bulk atoms, surface atoms have fewer bonds with each other at the free surface and require less energy to induce morphology change^12^,^21^,^33^. This results in a significant decrease in sintering and melting temperature as the size of particles is decreased to nanoscale due to the change in surface to volume ratio and cohesive energy of nanomaterials^12^,^21^,^33^,^34^,^35^.

The coalescence of Ag NF III was also investigated by differential scanning calorimetry (DSC) analysis (Fig. 3f). The ramping temperature was 5 °C min^−1^. Two exothermic peaks were observed at 122 and 144.5 °C which are significantly lower than the melting temperature of bulk silver (961.8 °C)^21^. It is interesting to note that the first sintering temperature (122 °C) observed in DSC analysis was higher than the deformation temperature (80 °C) observed in SEM analysis. It is possible that the coalescence behavior was too slow at 80 °C to induce a distinct exothermic peak in DSC analysis although the precise mechanism needs to be investigated further. Figure 3g shows an HRTEM image of coalesced petals of two Ag NF III particles. The lattice resolved HRTEM image of the marked square region shows a change in orientation of (111) planes at the merged border between two petals (Fig. 3h). The occurrence of double diffraction spots with similar intensity also indicated coalescence of two single-crystalline petals with rotated grain boundary (Fig. 3h inset)^36^. This demonstrated poly-crystalline nature of coalesced petals of Ag NFs, and a schematic representation of merged planes is provided in Fig. 3i.

## Ag NFs for Highly Conductive Inks

**Figure 4:**
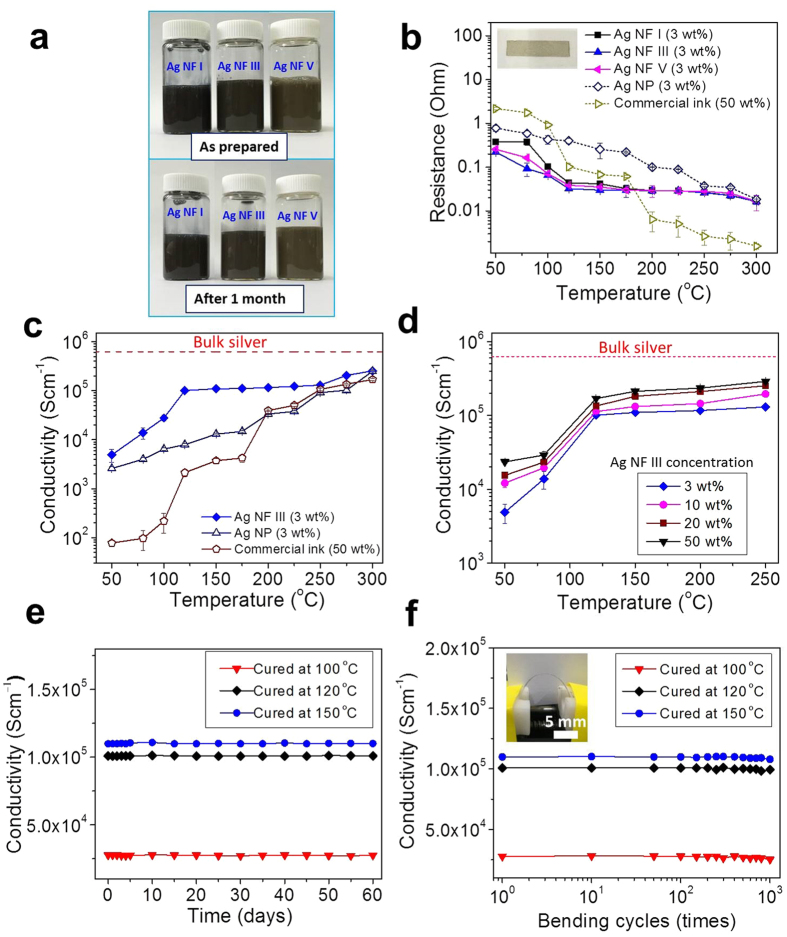
Ag NF inks. (**a**) Optical images of Ag NF inks (Ag NF = 3 wt%). (**b**) The resistance of ink pattern as a function of the curing temperature. An optical image of the specimen is provided in the inset (20 × 5 mm^2^). A commercial ink (Ag = 50 wt%) and a spherical silver nanoparticle ink (Ag NP, Ag = 3 wt% in HPMC solution) were also investigated. (**c**) The conductivity of specimens as a function of the curing temperature. (**d**) The effect of Ag NF III concentration on the conductivity of specimens. (**e**) The conductivity stability of Ag NF III specimens in ambient air environment. (**f**) The bending cyclability of Ag NF III specimens. The minimum bending radius was 5 mm (inset).

The excellent coalescence property of Ag NFs at low temperatures was employed to synthesize highly conductive inks. The conductive inks prepared using spherical silver nanoparticles, silver nanowires, or copper nanoparticles typically require high curing temperatures (>200 °C) to achieve high conductivity (~10^5^ Scm^−1^)^28^,^37^. This is not favorable for flexible or stretchable electronics which require soft polymeric substrates. Figure 4a shows water-based Ag NF inks, and a schematic of the synthesis process is provided in Supplementary Fig. 10. The concentration of Ag NFs was only 3 wt%, but it still provided high conductivity (~10^5^ Scm^−1^) as will be discussed shortly. Water was chosen as an environmentally-friendly solvent (96.40 wt%). Water-soluble viscoelastic HPMC was selected as a stabilizing agent (0.6 wt%)^38^,^39^. The hydroxyl groups in HPMC have affinity on silver^39^ and interacted with large surface of Ag NFs providing stable dispersion for more than one month (Fig. 4a, Ag NF = 3 wt%). The ink was untouched before taking the image after one month. The viscosities of Ag NF I, III, and V inks were 5.44, 3.12, and 2.01 mPa·s, respectively (ARES-G2 rheometer, shear rate = 100 s^−1^, temperature = 25 °C). The ink with smaller Ag NFs exhibited a higher viscosity, at the same weight concentration of 3 wt%, since the stronger particle-particle interaction was induced by a greater number of particles^40^.

The Ag NF ink pattern could be successfully formed on both flexible polyethylene terephthalate (PET) and rigid glass substrates. The glass transition and melting temperatures of PET were 78 and 255 °C. The PET substrate was used at lower curing temperatures (≤170 °C), and the glass substrate was employed at higher curing temperatures (>170 °C). Figure 4b compares resistance of the ink pattern formed by dropping 40 μl of Ag NF ink on a pre-defined mold (20 × 5 × 0.05 mm^3^). The ink was then air-dried for 12 hours followed by curing at different temperatures (30 min). Three specimens were prepared at each condition, and the average values and standard deviations are provided. A spherical silver nanoparticle ink (Ag:HPMC:water = 3:0.6:96.4 wt%) and a commercial silver nanoparticle ink (Ag = 50 wt%, Sigma Aldrich 796042, particle median diameter: ~70 nm) were also tested as controls. The spherical silver nanoparticle ink and Ag NF ink have the identical dispersant and solvent composition. The same amount (3 wt%) of spherical silver nanoparticles (~70 nm) were employed, instead of Ag NFs, for the spherical silver nanoparticle ink. The average film thickness was 865~878 nm for the patterns formed by the Ag NF ink and the spherical silver nanoparticle ink at the curing temperature of 120 °C. However, the identical volume of the commercial ink resulted in a greater thickness (12.9 μm) due to the greater silver concentration. Cross-sectional SEM images of Ag NF III specimens on PET substrates, before and after curing at 120 °C, are provided in Supplementary Fig. 11. The porosity and film thickness decreased after the curing due to the coalescence of Ag NFs. The resistance of the Ag NF specimens, measured by the four-point probe method, decreased with increasing temperature and stabilized at a curing temperature of 120 °C. Ag NF III (bud: ~400 nm, petal thickness: ~12 nm) provided minimum resistance among Ag NF inks. The increase in resistance for Ag NF V could be due to the thicker petals. The spherical silver nanoparticle ink resulted in a greater resistance compared with Ag NF inks. Surprisingly, the commercial ink with a greater silver concentration (50 wt%) provided greater resistance than the Ag NF ink (Ag NF = 3 wt%), in spite of the greater thickness of specimen, when the curing temperature was ≤175 °C. The active coalescence among Ag NFs was already achieved at 120 °C as discussed in Fig. 3, and a further decrease in resistance was small for Ag NF patterns at higher curing temperatures (>150 °C). In contrast, the commercial ink showed a gradual decrease in resistance with increasing curing temperature (50~300 °C) and provided minimum resistance only at ≥ 200 °C. Evidently, the high curing temperature (≥200 °C) is crucial for the commercial ink to induce effective connectivity among silver nanoparticles and realize low resistance.

Figure 4c compares the dimension-normalized conductivity of ink patterns. The conductivity of Ag NF III specimens dramatically increased at 80~120 °C reaching 1.008 × 10^5^ Scm^−1^ at 120 °C. The conductivities of the spherical silver nanoparticle ink (7925 Scm^−1^) and the commercial silver nanoparticle ink (2127 Scm^−1^) specimens were much lower at 120 °C. The conductivity of the Ag NF III specimen was two orders of the magnitude greater than that of the commercial ink specimen in spite of the significantly smaller silver concentration (3 vs 50 wt %). As discussed in Fig. 3, the active coalescence among Ag NFs was already achieved at 120 °C resulting in a high conductivity (>10^5^ Scm^−1^). Therefore, a further increment in conductivity (20, 434 Scm^−1^) over 150~250 °C was relatively small due to the saturation behavior. In contrast, the sintering was not effectively realized for the spherical silver nanoparticle ink at 120 °C which has the identical solvent and dispersant composition. The SEM observation also indicated that the active coalescence of spherical silver nanoparticles (~70 nm) was not realized at 120 °C (see Supplementary Fig. 9). The progressive sintering among spherical silver nanoparticles resulted in a relatively large increment in conductivity (79, 122 Scm^−1^) over 150~250 °C.

The thermal decomposition temperature of tripropylene glycol mono methyl ether-based solvent for the commercial ink was higher than water as shown in the thermogravimetric analysis (TGA, Supplementary Fig. 12a). However, it is important to note that the conductivity of specimens was measured after 12-h air-drying and additional 30-min curing (Fig. 4c). It is possible that the solvent was not completely removed at low curing temperatures. However, the solvent was dried at high curing temperatures (>100 °C) as shown in Supplementary Fig. 12b. The comparative TGA was carried out using powderized specimens after 12-h air-drying and 30-min curing at 120 °C. For the specimens made using Ag NF and spherical silver nanoparticle inks, the weight loss at 220~315 °C was due to the decomposition of HPMC. The observed relative concentration between HPMC and Ag NFs was similar to the initial mixture composition (HPMC:Ag NFs = 16.7:83.3 wt%). The relative concentration of silver was even higher (remaining dispersant: Ag = 2.4:97.6 wt%) for the commercial ink. Optical microscopic images confirmed the dried commercial ink specimen (Supplementary Fig. 12b inset). This indicated that the active coalescence of Ag NFs resulted in the higher conductivity (1.008 × 10^5^ Scm^−1^) compared with the commercial ink specimen (2127 Scm^−1^) at the curing temperature of 120 °C. Besides, the conductivity of the Ag NF ink specimen was significantly higher than that of the self-made spherical silver nanoparticle ink specimen (7925 Scm^−1^) demonstrating the excellent coalescence characteristics of Ag NFs.

The conductivity of Ag NF III specimens could be further increased (1.694 × 10^5^ Scm^−1^ at 120 °C) by increasing the concentration of Ag NFs (50 wt%) as shown in Fig. 4d. The dispersion stability decreased as the concentration of Ag NFs was increased to 50 wt%. The concentration of HPMC was kept the same as the Ag NF ink (Ag NF = 3 wt%). Ag NFs settled down after a few days. However, they could be easily redispersed after slight hand shaking (see Supplementary Fig. 13). The conductivity of Ag NF III specimens, cured at 3 different temperatures, was invariant over 60 days in ambient air environment demonstrating long-term stability (Fig. 4e). Figure 4f shows bending cycleability of Ag NF III specimens on flexible PET substrates. The minimum bending radius was 5 mm. The change in conductivity was negligible over 1000 cycles demonstrating excellent flexibility.

Numerous efforts have been reported to reduce sintering temperature of silver particles. These include silver nanoparticles with decreased size^5^,^6^,^33^,^34^,^35^, dendrite structure^2^, hybrids of silver nanoparticles and nanowires^12^, surfactant-free synthesis method^41^, and employment of thermally labile precursors^42^. Here we developed a simple scalable surfactant-free synthesis method of Ag NFs with high specific surface area. The coalescence of hierarchically-structured nanoscale petals at a low curing temperature (~120 °C) dramatically increased connectivity among Ag NFs resulting in a greater conductivity than spherical silver nanoparticles.

## Conclusion

A novel synthesis method of Ag NFs was developed. The synthesis of Ag NFs was scalable with a high yield of >99.5%. The preferential affinity of citrate molecules on (111) surface of silver enabled spontaneous anisotropic growth of Ag NFs in aqueous solution. The bud size (250~580 nm) could be controlled by the concentration of ascorbic acid, and single-crystalline petals (thickness = 9~22 nm) were protruded from the surface. The unique flower-shaped structure resulted in coalescence of petals starting from the curing temperature of 80 °C, and striking morphology change was observed at 120 °C. This excellent coalescence behavior enabled the construction of percolation pathways among Ag NFs which was practically employed in conductive ink. The concentration of Ag NFs in ink was only 3 wt%. Nevertheless, it provided two orders of magnitude greater conductivity (1.008 × 10^5^ Scm^−1^), at a low curing temperature of 120 °C, compared with the silver nanoparticle ink with a much higher silver concentration (50 wt%). The low curing temperature, excellent dispersibility in ink, and high conductivity at a low concentration may provide an opportunity for Ag NFs for applications in printable and flexible electronics.

## Methods

### Synthesis of Ag NFs

The Ag NFs were synthesized in three steps. Firstly, aqueous solutions of silver nitrate (High purity chemicals, 3692741, 0.3 M, 50 ml) and ammonium citrate dibasic (Sigma Aldrich, 09833, 0.15 M, 50 ml) were mixed for 2 minutes at 50 °C resulting in whitish precipitate of silver citrate complex^22^. In the second step, an aqueous ammonia solution (Samchun Chemicals, A0628, 14.8 M) was added drop by drop until the solution became colorless, and an aqueous boric acid solution was added (Sigma Aldrich, B6768, 0.5 M, 50 ml)^17^,^43^. Finally, an aqueous L-ascorbic acid solution (Sigma Aldrich, A5960, 0.1~1 M, 50 ml) was added^15^,^18^,^25^. The color of the solution immediately turned into dark brown indicating synthesis of Ag NFs. After completing the reaction in 15 minutes, Ag NFs (~1.6 g) were collected by vacuum filtration (PTFE membrane, 0.2 μm) and repeatedly rinsed with deionized water. Ag NFs were then dried in an oven at 40 °C for 12 h. The synthesis could be upscaled by increasing the amounts of reagents.

### Preparation of Ag NF ink

Firstly, HPMC (Sigma Aldrich, H3785, 60 mg) was dissolved in 9.64 g of deionized water. Ag NFs (300 mg) were then added and tip-sonicated for 20 min (560 W). The Ag NF concentration was 3 wt%. The Ag NF ink (40 μl) was dropped onto a pre-defined mold (20 × 5 × 0.05 mm^3^) constructed by Scotch tape on a PET or glass substrate. The ink was cured at 50~300 °C for 30 min in ambient air environment after air drying for 12 h. As a control, spherical silver nanoparticles (Sigma Aldrich, 484059, average diameter measured from SEM images: ~70 nm, 300 mg) were also dispersed in HPMC (60 mg)-dissolved water (9.64 g) by tip-sonication (560 W, 20 min). A commercial silver nanoparticle ink was also tested (Ag = 50 wt%, Sigma Aldrich, 796042).

### Characterization

The morphology of Ag NFs was characterized by SEM (JOEL, JSM-7600F) and HRTEM (JOEL, JEM-2100F). The crystal property was examined by powder X-ray diffraction analysis (RIGAKU, XRD-2500V/PC). The specific surface area of Ag NFs was characterized by BET analysis (BEL Japan, BELSORP-mini II), and light absorption was characterized by UV-Vis spectroscopy (Shimadzu, UV-Vis-NIR spectrophotometer UV3600). DSC (SEICO, DSC 7020) analysis was carried out under nitrogen atmosphere.

The resistance of ink patterns was measured using a four-point probe in-line method with a current source (Keithly 6221) and a nanovoltameter (Keithely 2182A)^44^. The distance between probes was 1 mm. The volume resistivity was then calculated using the formula 
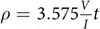
, where *V* is the measured voltage, *I* is the supplied current, and *t* is the thickness of the pattern^5^,^28^. The thickness of specimens was measured by an alpha step surface profiler (KLA Tencor). The thickness of the periphery of specimens was not uniform, which was in direct contact with the Scotch tape mold, and the averaged thickness of flat central region was used to calculate conductivity.

## Additional Information

**How to cite this article**: Muhammed Ajmal C. *et al*. Hierarchically-structured silver nanoflowers for highly conductive metallic inks with dramatically reduced filler concentration. *Sci. Rep.* **6**, 34894; doi: 10.1038/srep34894 (2016).

## Supplementary Material

Supplementary Information
