# Supplementary material for: Hierarchically-structured silver nanoflowers for highly conductive metallic inks with dramatically reduced filler concentration
Source: Sci Rep. 2016 Oct 7;6:34894. doi: 10.1038/srep34894 (PMC5054671; doi:10.1038/srep34894)
Supplement: Supplementary Information [file srep34894-s1.pdf]

SUPPLEMENTARY INFORMATION

Hierarchically-structured silver nanoflowers for highly conductive metallic inks with dramatically reduced filler concentration

C. Muhammed Ajmal[1], K.P. Faseela[1], Swati Singh[1], and Seunghyun Baik[2,3]*

[1] Department of Energy Science, Sungkyunkwan University, Suwon, 16419, Republic of Korea

[2] School of Mechanical Engineering, Sungkyunkwan University, Suwon, 16419, Republic of Korea

[3] Center for Integrated Nanostructure Physics, Institute for Basic Science (IBS), Suwon, 16419, Republic of Korea

***e-mail: sbaik@me.skku.ac.kr**

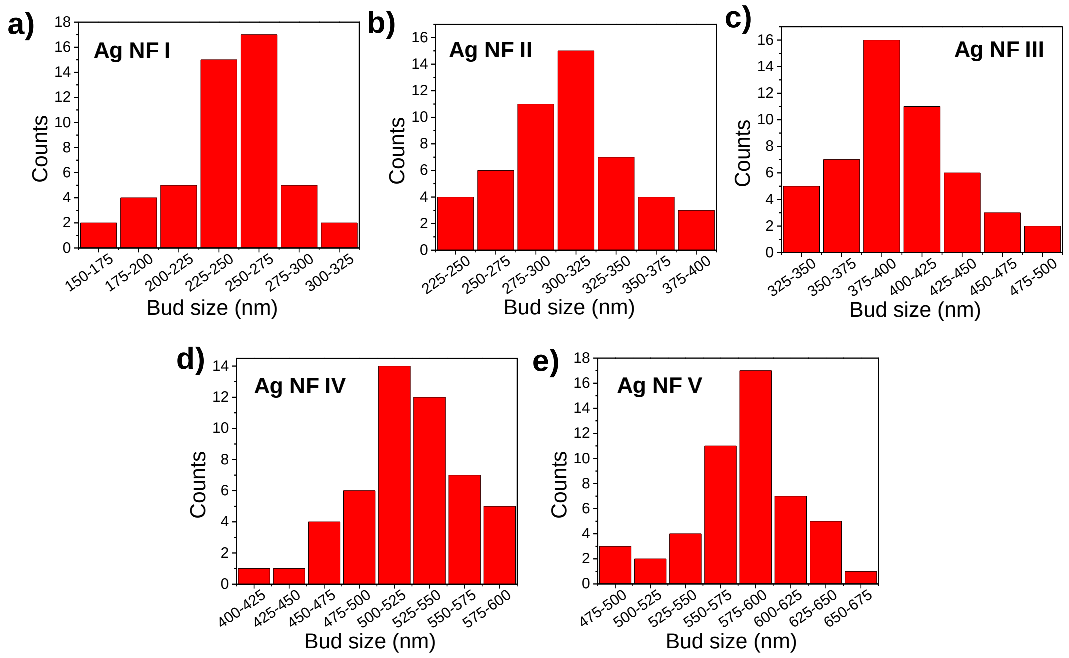

Supplementary Figure 1. The bud size distributions of Ag NFs. The total sum of counts was 50 for each type. (a) Ag NF I. (b) Ag NF II. (c) Ag NF III. (d) Ag NF IV. (e) Ag NF V.

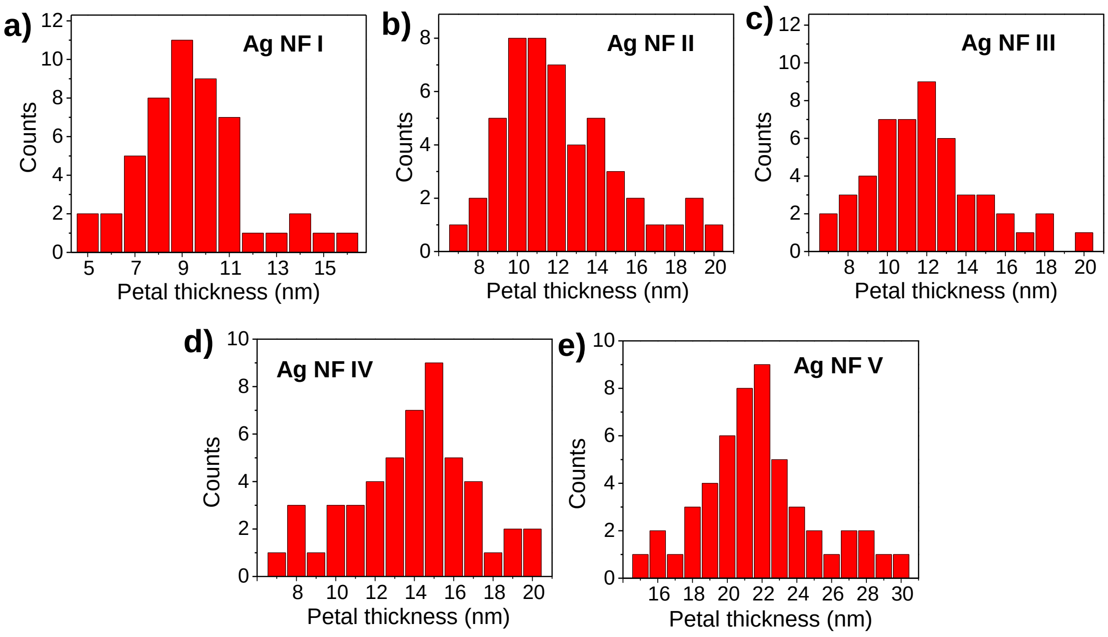

Supplementary Figure 2. The petal thickness distributions of Ag NFs. The total sum of counts was 50 for each type. (a) Ag NF I. (b) Ag NF II. (c) Ag NF III. (d) Ag NF IV. (e) Ag NF V.

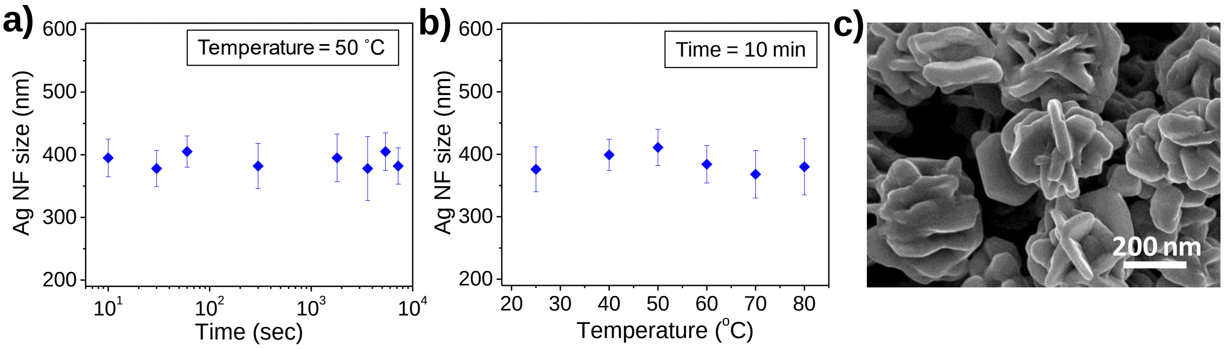

Supplementary Figure 3. The effects of synthesis time (a) and temperature (b) on the bud size of Ag NF III. (c) An SEM of Ag NF III synthesized at 100 ˚C for 15 min.

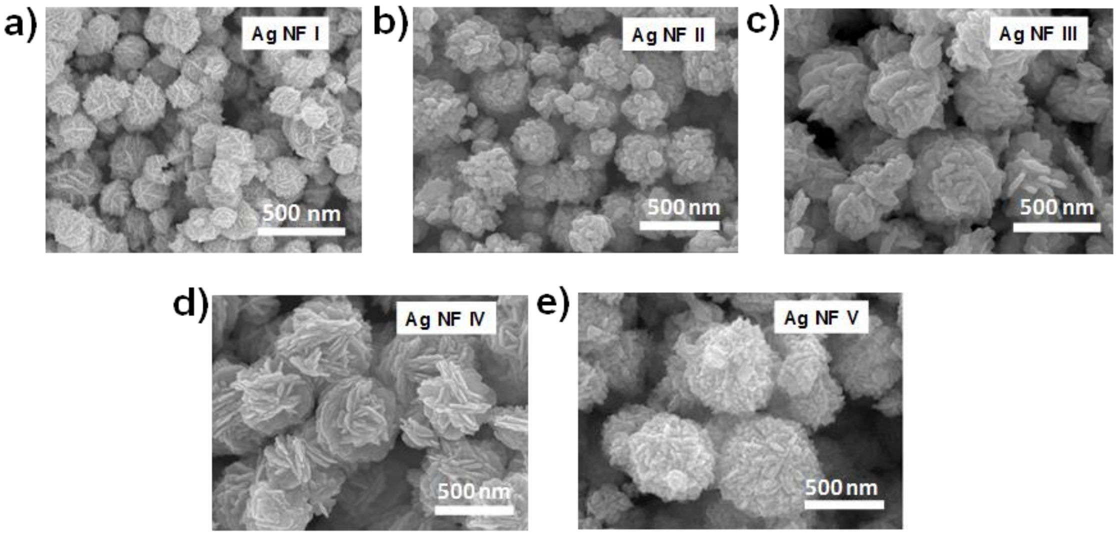

Supplementary Figure 4. SEM images of Ag NFs synthesized using different ascorbic acid concentrations. (a) Ag NF I (0.1 M). (b) Ag NF II (0.15 M). (c) Ag NF III (0.3 M). (d) Ag NF IV (0.5 M). (e) Ag NF V (1 M).

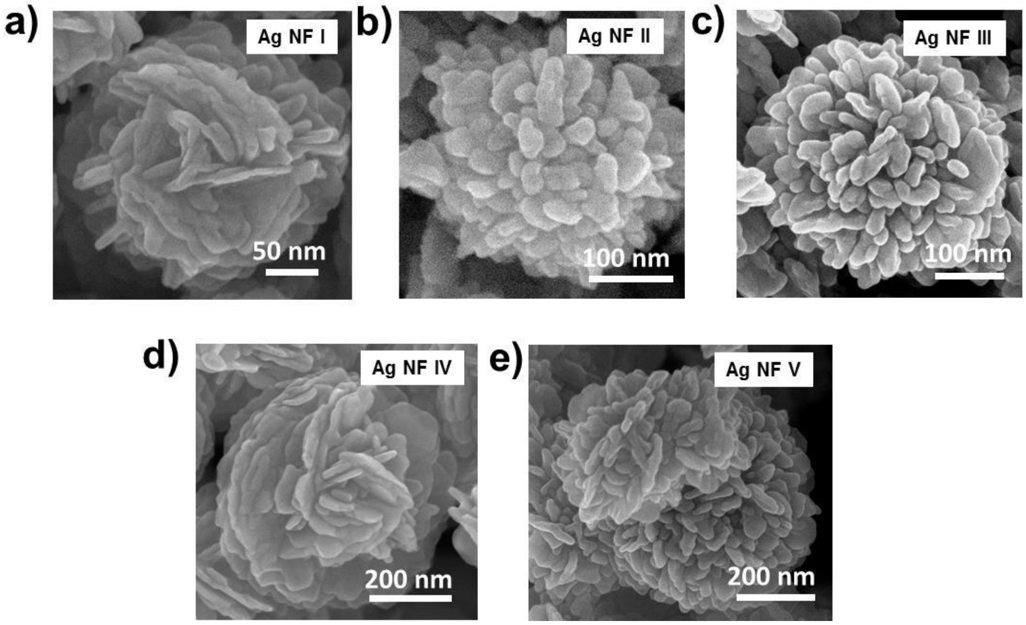

Supplementary Figure 5. Magnified SEM images of Ag NFs. (a) Ag NF I. (b) Ag NF II. (c) Ag NF III. (d) Ag NF IV. (e) Ag NF V.

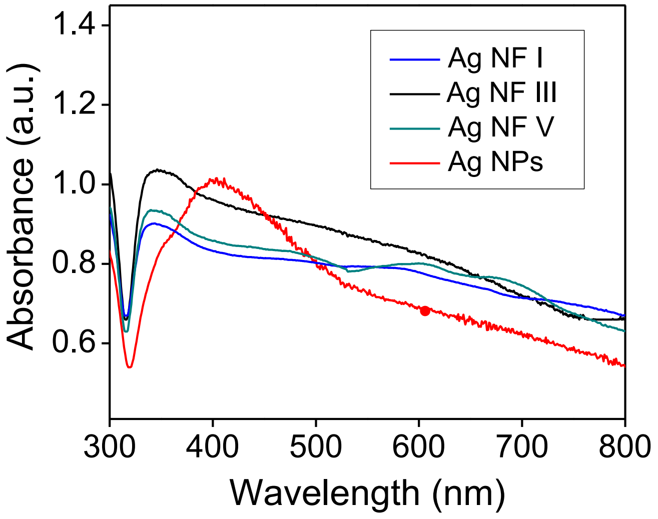

Supplementary Figure 6. UV-Vis absorption spectra of Ag NFs and spherical silver nanoparticles (Ag NPs).

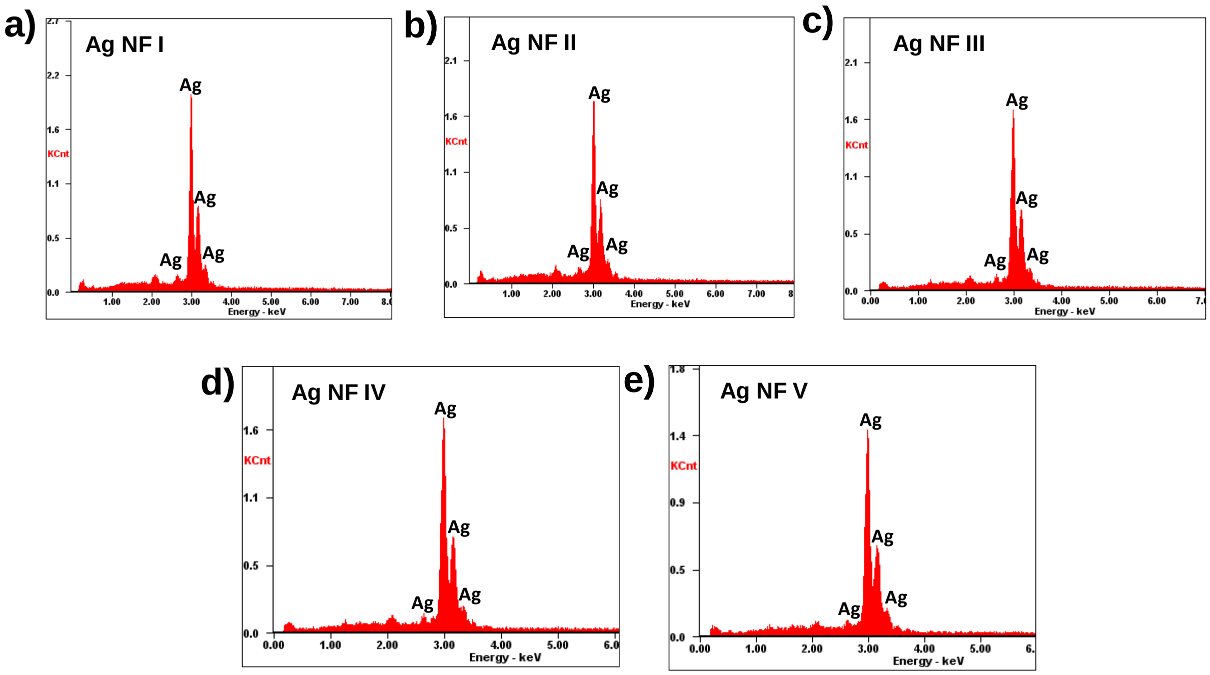

Supplementary Figure 7. Energy-dispersive X-ray analysis of Ag NFs. (a) Ag NF I. (b) Ag NF II. (c) Ag NF III. (d) Ag NF IV. (e) Ag NF V.

**Curing time**	**Curing temperature**	
	60 ˚C	80 ˚C
60 min	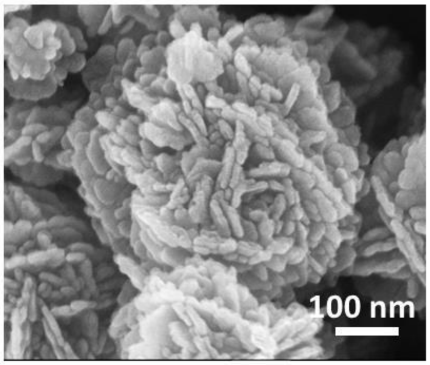	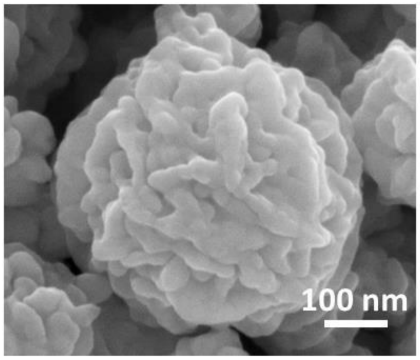
90 min	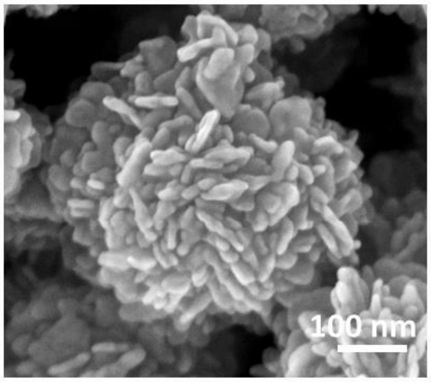	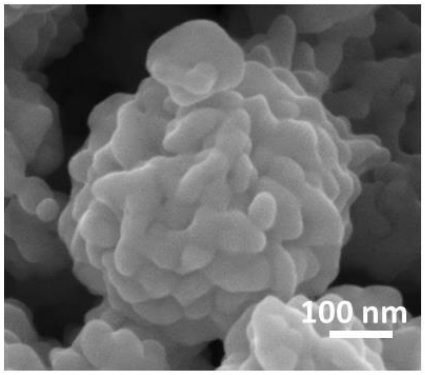
120 min	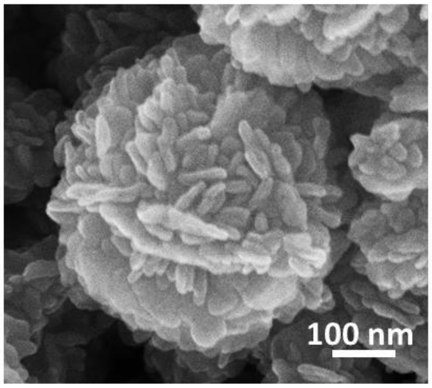	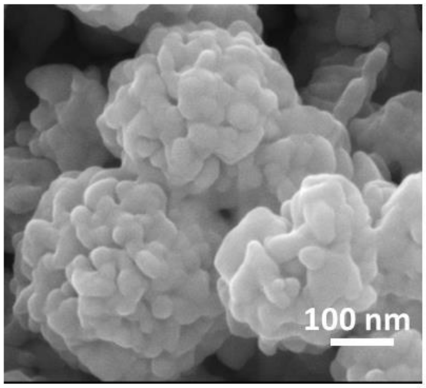

Supplementary Figure 8. SEM images of Ag NFs cured at 60 or 80 ˚C. The images show different Ag NFs in the powder mixture.

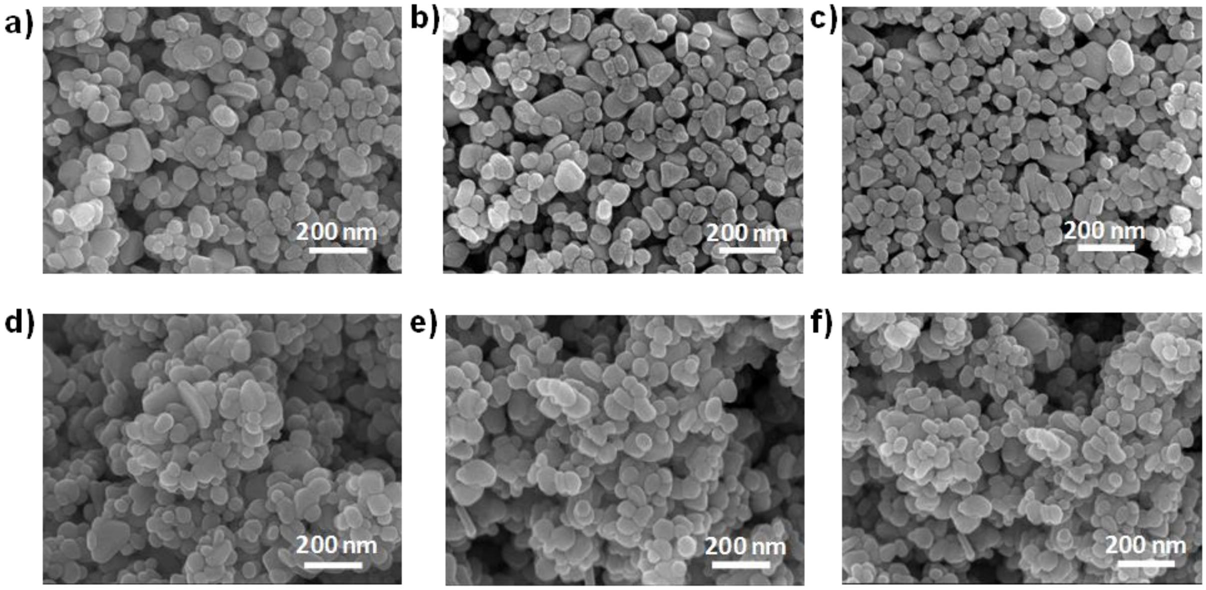

Supplementary Figure 9. SEM images of spherical silver nanoparticles cured at different temperatures for 30 min. The average diameter measured from SEM images was ~70 nm. (a) Room temperature (~22 ˚C). (b) 60 ˚C. (c) 80 °C. (d) 100 °C. (e) 120 °C. (f) 150 °C.

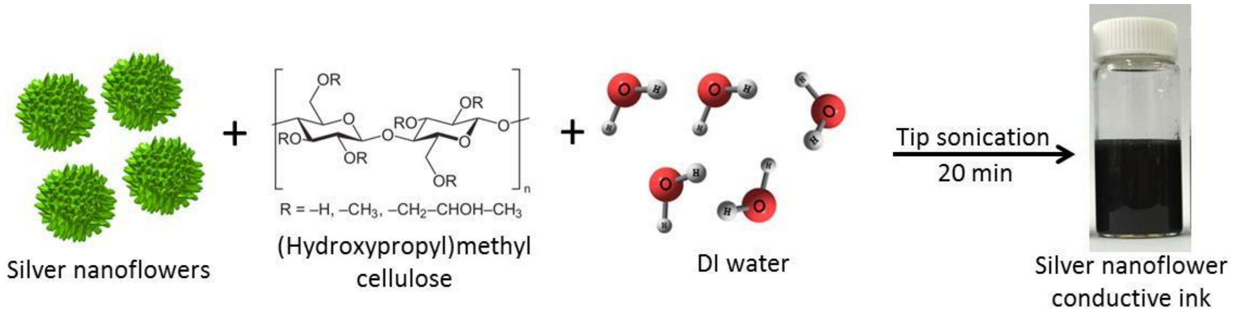

Supplementary Figure 10. The preparation process of Ag NF ink.

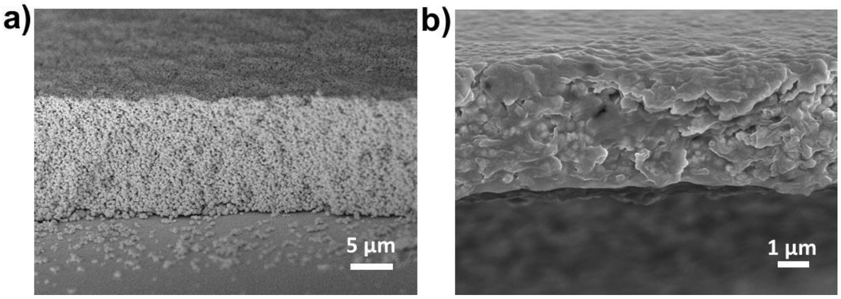

Supplementary Figure 11. Cross-sectional SEM images of the Ag NF III ink (Ag = 3 wt%) pattern before (a) and after (b) the curing at 120 ˚C for 30 min. The volume of the ink was increased to 120 µl to increase thickness of the pattern for better cross-sectional imaging.

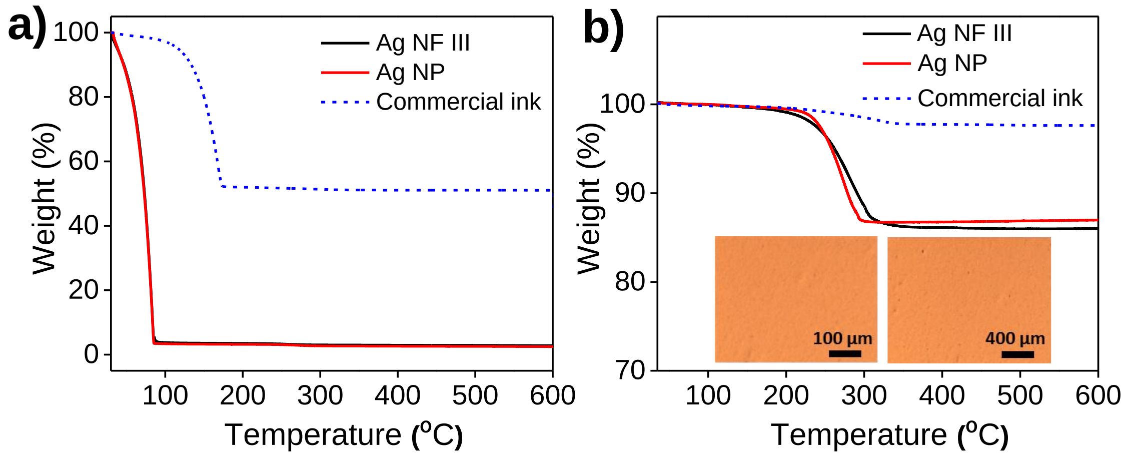

Supplementary Figure 12. (a) The comparative thermogravimetric analysis (TGA) of the Ag NF III ink (3 wt%), spherical silver nanoparticle ink (Ag NP, 3 wt%), and commercial ink (50 wt%). (b) The specimens after 12-h air drying and 30-min curing at 120 °C were powderized, and TGA was carried out using powders. The inset images show low- and high-magnification optical microscopic images of the commercial ink specimen after 12-h air drying and 30-min curing at 120 °C.

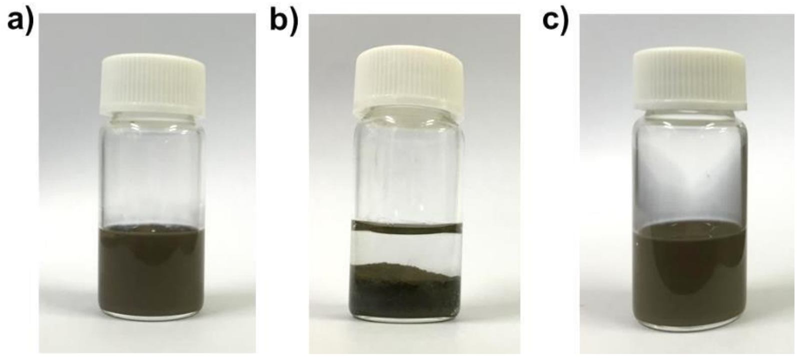

Supplementary Figure 13. Optical images of Ag NF ink (Ag NF = 50 wt%). (a) As prepared. (b) After 3 days. (c) Redispersed Ag NF ink after slight hand shaking.
